# Myco-Synthesized Selenium Nanoparticles as Wound Healing and Antibacterial Agent: An In Vitro and In Vivo Investigation

**DOI:** 10.3390/microorganisms11092341

**Published:** 2023-09-19

**Authors:** Heba El-Sayed, Mostafa Y. Morad, Hana Sonbol, Olfat A. Hammam, Rehab M. Abd El-Hameed, Rania A. Ellethy, Amina M. Ibrahim, Marwa A. Hamada

**Affiliations:** 1 Botany and Microbiology Department, Faculty of Science, Helwan University, Helwan 11795, Egypt; drhebaelsayed39@gmail.com (H.E.-S.); roby1432000@gmail.com (R.M.A.E.-H.); marwa.hamada@science.helwan.edu.eg (M.A.H.); 2 Zoology and Entomology Department, Faculty of Science, Helwan University, Helwan 11795, Egypt; myame_mostafa@yahoo.com; 3 Department of Biology, College of Science, Princess Nourah Bint Abdulrahman University, P.O. Box 84428, Riyadh 11671, Saudi Arabia; 4 Pathology Department, Theodor Bilharz Research Institute, Giza 12411, Egypt; totoali@hotmail.com; 5 Chemistry Department, Faculty of Science, Helwan University, Ain Helwan, Cairo 11795, Egypt; raniaellethy@science.helwan.edu.eg; 6 Medical Malacology Department, Theodor Bilharz Research Institute, Giza 12411, Egypt; aminamd.ibrahim@yahoo.com

**Keywords:** selenium nanoparticles, wound healing, antibacterial, *Staphylococcus aureus*, pro-inflammatory cytokines, docking study

## Abstract

Bacterial-associated wound infections are an obstacle for individuals and the medical industry. Developing versatile, antibiotic-free therapies helps heal wounds more quickly and efficiently. In the current study, fungal metabolites were employed as a reducing agent in fabricating selenium nanoparticles (SeNPs) for improved antibacterial and wound healing properties. Utilizing UV-visible spectroscopy, dynamic light scattering (DLS), zeta potential, X-ray diffraction (XRD), and electron microscopic examination, the properties of the synthesized nanoparticles were extensively evaluated. Myco-synthesized SeNPs demonstrated strong antibacterial activity against *Staphylococcus aureus* ATCC 6538 with a minimum inhibitory concentration of 0.3125 mg/mL, reducing cell number and shape distortion in scanning electron microscope (SEM) images. SeNPs’ topical administration significantly reduced wound area and healing time, exhibiting the least bacterial load after six days compared to controls. After six and 11 days of treatment, SeNPs could decrease proinflammatory cytokines IL-6 and TNF-α production. The histopathological investigation showed a healed ulcer with moderate infiltration of inflammatory cells after exposing mice’s skin to SeNPs for six and 11 days. The docking interaction indicated that SeNPs were highly efficient against the IL-6 and TNF-α binding receptors. These findings imply that myco-fabricated SeNPs might be used as topically applied antimicrobial agents for treating skin infections and wounds.

## 1. Introduction

Wound infection is marked by the invasion of bacteria and other microbes, which may result in the delaying of wound healing or, worse, cause deterioration. Bacterial contamination of the skin, other body parts, and external environments is the primary cause of many wound infections [1]. Healthy skin has three layers: the outer epidermis, the dermis, and the fatty subcutaneous layer, which serves as a protective barrier [1]. However, when impaired, the loss of the outer epidermal barrier, along with protein and lipid denaturation, leads to an ideal environment for bacterial growth [2,3]. As a result, the infection activates the immune system, causing inflammation and delaying the healing process. Although most wound infections heal on their own, serious untreated wounds or insufficient treatment may last and become potentially life-threatening. The main objective of wound treatment is to limit or eliminate infectious agents while supporting wound healing. Wound healing is one of the most difficult biological processes, which includes four stages: homeostasis, inflammation, proliferation, and maturation of new tissue [4]. Infections cause a delay in the healing process of wounds. The skin infected with *Staphylococcus aureus* is considered drug-resistant and categorized as one of the five known hospital-acquired infections [3]. When bacteria enter the body through wounds, they colonize deep into the body, which may affect the healing process and hasten natural healing. Proinflammatory cytokines, which influence immune cell behavior during epithelialization, are among the first weapons released in response to skin lesions. Wound infection may prolong the inflammatory phase. Tumor necrosis factor (TNF), interleukin (IL)-1, IL-6, and IL-17 are the key proinflammatory cytokines that contribute to the inflammatory phase of wound healing [5]. These infections will affect the clotting process that occurs normally in wounds. As a result, the angiogenesis process will be slowed. It becomes critical to treat these infections to heal the wound [6].

Many researchers have been working on exploring new strategies for wound healing, including antibacterial and antifungal products [7]. To date, one of the most biologically efficient wound healing strategies is incorporating nanotechnology products [7]. Because of their distinctive microscopic size and surface effects, as well as tunable physiochemical properties, nanomaterials with diameters of less than 100 nm have been widely employed to inhibit infectious microbes and improve wound recovery [8,9]. To improve antibacterial efficacy and decrease bacterial drug resistance, metal nanoparticles with fundamental antibacterial properties have been widely used. Their high ratio of surface-to-volume features allows for adequate contact and interaction with bacteria [10].

Selenium nanoparticles (SeNPs) are a new multifunctional therapy recently developed and synthesized [11,12]. Selenium is required by almost all organisms; however, beneficial and hazardous selenium levels exist. SeNPs carry all of the physiological activities of selenium, have reduced toxicity, and have better biological activity, making it the ideal selenium type for managing selenium’s narrow application dose range [13,14,15,16]. At the same time, SeNPs have diverse biological actions in vivo, including antioxidant, antibacterial, antiviral, and anticancer properties [17,18]. To now, no bacterial resistance has been detected in antibacterial studies on SeNPs, making SeNPs an excellent candidate for treating infectious wound healing [11]. SeNPs have been shown to suppress infections caused by human pathogenic bacteria such as *S. aureus*, *L. monocytogenes*, *E. faecalis*, *S. agalactaie*, and *B. cereus* [19]. According to Serov et al. [20], the antibacterial action of SeNPs can be explained by various mechanisms, including protein degradation brought on by selenium nanoparticles’ antibacterial effects. Slow emission of selenium ions from nanoparticle surfaces may interact with the -SH, -NH, or -COOH functional groups of proteins and enzymes, resulting in the loss of their tertiary and quaternary structure and functionalities. The natural processes for transporting ions and nutrients through cell walls are deactivated by SeNPs, which prevents the cell from performing its essential functions. Hyperproduction of reactive oxygen species (ROS), membrane potential alteration, and reduction of internal ATP. Degradation of the cell membrane’s integrity and inhibition of the dehydrogenase enzyme’s function. Inhibition of bacterial adhesion and production of bacterial films. Bactericidal effect of photocatalysis.

The current study attempted to evaluate the antibacterial activity of myco-synthesized SeNPs against *Staphylococcus aureus* in vitro and in vivo, as well as to investigate their potential as a wound-healing agent.

## 2. Materials and Methods

### 2.1. The Fungal Culture

*Penicillium chrysogenum* MZ945518 was isolated from the Mediterranean coast in Alexandria, Egypt, and described using molecular techniques as previously explained by Morad et al. [21].

### 2.2. Green Synthesis of SeNPs

SeNPs were prepared and characterized according to Hussein et al. [22] with some modifications as follows: first, *P. chrysogenum* was regularly sub-cultured on PDA media and incubated at 28 °C for 5 days; then, the fungal discs were transferred to a potato dextrose broth medium and statically incubated for a week at 25 °C. After that, the culture was centrifuged at 10,000 rpm for 10 min (Sigma3-16k, Sigma Laborzentrifugen GmbH, Osterode am Harz, Germany) to separate the supernatant from the mycelium. Finally, 1 mL of 3 mM sodium selenite was added to 10 mL of the culture supernatant, and the whole reaction was incubated at 40 °C for 30 min. Following that, a reddish hue appeared, showing the complete synthesis of the SeNPs. The formed nanoparticles were centrifuged for 10 min at 10,000 rpm, and the SeNP pellet was collected. The myco-fabricated SeNPs were then purified by mixing with double-distilled water and centrifuged thrice for 10 min each at 10,000 rpm. The resulting nanoselenium was dried for 48 h at 60 °C and then stored for later use.

### 2.3. Characterization of SeNPs

The color change in the sodium selenite solution incubated with *P. chrysogenum* culture supernatant was clearly detected using UV-visible spectroscopy. The absorbance of the nanoparticle solution and the control sample was detected between 300 and 800 nm using a UV-visible spectrophotometer (PerkinElmer Life and Analytical Sciences, CT, Ohio, USA). To determine the size, shape, aggregation, and morphological characteristics of the SeNPs, transmission electron microscopy (TEM) images of high quality were collected using a TEM (HR-TEM; JEOL 2100, Japan). The FTIR spectra of the control medium and SeNP solutions were recorded using FTIR (PerkinElmer, Ohio, USA). All measurements were performed between 400 and 4000 cm^−1^. Dynamic light scattering (DLS) measurements using Zetasizer (Zetasizer Nano ZN, Malvern Panalytical Ltd., Malvern, UK) at an angle equal to 173° and a temperature of 25 °C were used to assess the particle size distribution and zeta potential of SeNPs. By measuring the polydispersity index (PDI), DLS analysis also revealed further details regarding the homogeneity of the NPs solutions. X-ray diffraction (XRD) analysis (Bruker D8 DISCOVER Diffractometer, USA) was used to assess the crystallinity of the myco-fabricated SeNPs.

### 2.4. Antibacterial Efficacy of SeNPs against Staphylococcus aureus In Vitro

#### 2.4.1. Minimum Inhibitory Concentration (MIC) Estimation

The antibacterial activity of the biosynthesized SeNPs was tested against the reference bacterial strain *Staphylococcus aureus* ATCC 6538. The test was employed using the broth microdilution assay described by [23,24], with the following minor modifications: to make stock solutions, 1 mL of sterilized water was mixed with 2.5 mg of selenium nanoparticles. 400 µL of stock solution was applied to the first column of a microtiter plate. In each well (from 2 to 12), 200 µL of sterile tryptic soy broth (TSB) was added. Through transferring 200 µL from the first to the tenth well, a two-fold dilution was achieved. Fifty µL of the reference bacterial strain carrying 5 × 10^8^ CFU/mL (OD ~0.1 at 625 nm) was added to each well except the last one used as a blank. Then, the microtiter plate was incubated for 24 h at 37 °C. The data were read at 630 nm employing a ChroMate 4300 (Awareness Technology, Inc., Palm City FL, USA) Elisa reader.

#### 2.4.2. Scanning Electron Microscopy (SEM) of SeNP-Treated and Untreated *S. aureus* Cells Morphology

According to Lemos et al. [25], the morphological examination of *S. aureus* was employed to document the morphological integrity changes before and after treatment with SeNPs by SEM. Bacterial SEM imaging was implemented after cell exposure to SeNPs (in tryptic soy broth) for 24 h and shaking incubation at 37 °C using untreated *S. aureus* as a control. Both treated and untreated cells were collected in a 1.5 mL Eppendorf tube containing 2% glutaraldehyde in 1X phosphate buffer solution (1X PBS, pH 7.4). After that, samples were transferred to be processed as described by [26] and examined using a JEOL scanning electron microscope (JEOL GM 5200, Tokyo, Japan) in the Experimental Research Station at Cairo University’s Faculty of Agriculture in Giza, Egypt.

### 2.5. Mouse Model of Wound Infection

All animal protocols followed Helwan University’s instructions for the Care and Use of Laboratory Animals, which were approved by the university’s Animal Ethics Committee and assigned the study ethics number [HU-IACUC/Z/MY3107-31].

#### 2.5.1. Acquisition of Animals

Six-week-old male mice (~20 g) were purchased from the animal house of Theodor Bilharez Institute, Egypt, and were acclimatized in the laboratory for seven days. Mice were fed Purina chow (20% protein) and drank tap water.

#### 2.5.2. Excision Wound Development

For applying full-thickness excisional skin wounds, the mice were anesthetized using Diethyl ether. The back hair was shaved, cleaned, and disinfected before the experiment. In the top back of the mice, a sterile puncher created an oval full-thickness wound with a diameter of 3 mm.

#### 2.5.3. Groups and Treatment

**Figure 1:**
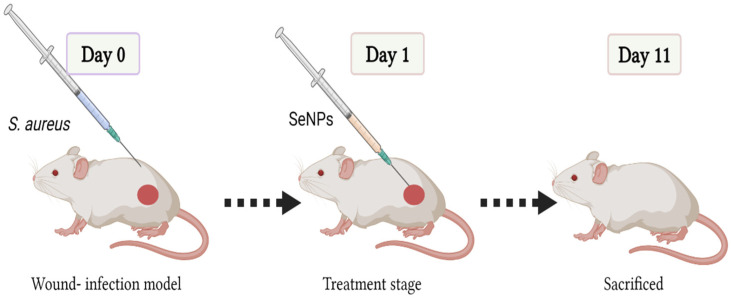
Schematic representative diagram for the wound-healing and antibacterial therapeutic process in vivo.

Three groups of mice (n = 10 per group) were housed individually, fed ad libitum, and allowed free water supply (Figure 1). Group I (negative control): full-thickness excisional skin wound mice treated with phosphate buffer saline (PBS) only; Group II (positive control): full-thickness excisional skin wound mice treated with gentamycin cream (Gentamicin 0.1%); Group III (treated group): full-thickness excisional skin wound mice treated with 20 µL of SeNPs (125 µg/mL). For 11 days, the treatments were given topically to the skin wound once a day.

### 2.6. Macroscopic Estimation of Wound Size

From the first day after therapy, daily wound photos were obtained. The wound area was calculated by drawing a circle across the wound’s border and estimating the distance along the wound bed [27]. A fine ruler was placed at the level of the wound and used to calibrate each image during measurement analysis to determine the magnification of the photographs.

### 2.7. Bacterial Load Determination

The bacterial load in untreated (negative control) and treated wounded tissues (positive control and SeNP treatment) were determined according to [28] with minor modifications. On day zero of the in vivo experiment, post bacterial inoculation, experimental mice’s infected back wounds were removed for bacterial culture of the different groups (positive control, negative control, treated with SeNPs at 0.3125 mg/mL) were collected using a disposable medical scalpel in sterile 1X phosphate buffer saline (PBS) and gently vortexed for 5 min. The three samples were serially diluted, and then 100 µL of each concentration was swabbed on the Muller Hinton Agar (MHA) media. Finally, the culture was kept for 24 h at 37 °C, and colony enumeration was performed the next day. In addition to zero day, the bacterial load was investigated on the sixth day.

### 2.8. Histopathological Studies

Autopsy samples were taken from the mice’s skin under investigation and controls on the sixth day of treatment and the end of the experiment, then fixed in formalin (10%) for 24 h. Alcohol was used in a serial dilution process to remove moisture from the samples; then, they were washed in xylene and embedded in paraffin for 24 h at 56 °C in a hot air oven. Tissue paraffin blocks were cut to a thickness of 4 µm using slide microtomes. Tissue samples were taken, deparaffinized, and stained with hematoxylin, eosin (H&E), and Masson’s trichrome for histopathological analysis under a light microscope [29,30].

### 2.9. Detection of Inflammatory Mediators

To detect inflammatory cytokines, an enzyme-linked immunosorbent test (ELISA) was utilized. Small sections of the injured skin were removed and washed in ice-cold phosphate-buffered saline (PBS 0.01 M and PH = 7.4) to remove all the blood. Homogenization of frozen tissue pieces in PBS (1 g/9 mL) was done using a glass homogenizer. To further break down the cells, the homogenate was sonicated using an ultrasonic cell disrupter (BioLogics, Inc., 150V/T, VA, USA). Finally, the homogenate was centrifuged for 5 min at 5000× *g*, and then the supernatant was kept at −80 °C for further assays. IL-6 and TNF-α cytokines were measured by a commercial ELISA kit following the manufacturer’s instructions (Elabscience, Houston, TX, USA). Briefly, the micro-ELISA plate was pre-coated with an antibody specific to IL-6 and TNF-α. Biotinylated detection antibodies specific for both cytokines and an Avidin-Horseradish Peroxidase (HRP) conjugate were added to each well after samples/standards had been added and then incubated. Using biotinylated detection antibodies, the IL-6 and TNF-α optical densities were determined. The optical density (OD) level correlated with the concentrations of both cytokines.

### 2.10. In-Silico Docking Interaction Study

The effect of SeNPs on the proinflammatory cytokines IL-6 and TNF-α was studied in silico. Using Protein Data Bank (PDB), the structure of these two cytokines was obtained and encoded (ID: 1alu) for human IL-6 [31] and (ID: 2az5) for human TNF-α [32]. The molecular docking was done with the help of the MOE program (MOE 2014.09). The energy of the ligand, SeNPs, was minimized, the proper sequence of both cytokines was chosen, followed by protonating, and finally, the partial charges were calculated.

### 2.11. Statistical Analysis

GraphPad Prism 9.0 (LLC) was the program used for the statistical analysis. Means were statistically compared using one-way ANOVA and Tukey’s post hoc test. The data were presented as the mean ± SD. It was regarded as significant if the probability level was less than 0.05.

## 3. Results

### 3.1. SeNPs Myco-fabrication and Characterization

**Figure 2:**
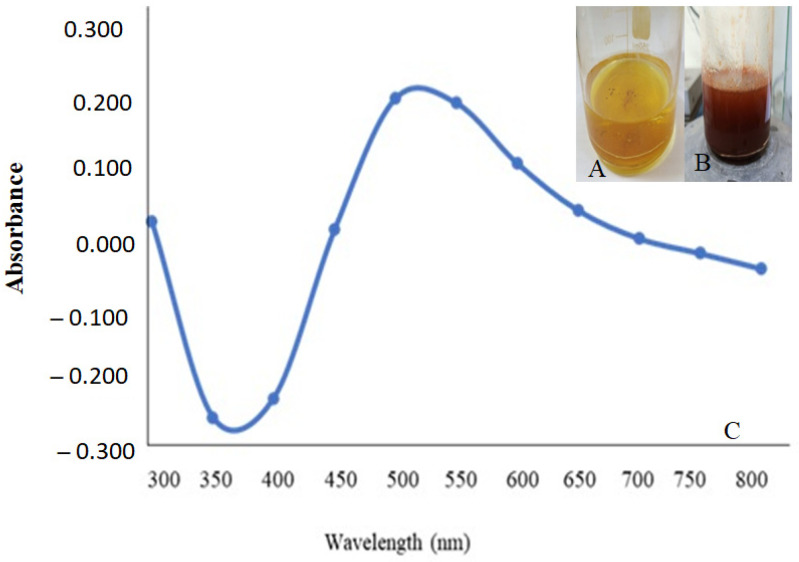
Myco-fabrication of SeNPs. (**A**) Culture filtrate, (**B**) SeNPs color, and (**C**) UV-visible characterization.

In the current research, *P. chrysogenum* MZ945518 culture filtrate metabolites were demonstrated to efficiently reduce Se ions and produce SeNPs. Adding a Na_2_SeO_3_ solution caused the fungal filtrate to transform from yellow to ruby red, indicating the presence of SeNPs, as illustrated in Figure 2A,B. The UV-visible absorbance of the SeNP particles was measured at 520 nm (Figure 2C).

**Figure 3:**
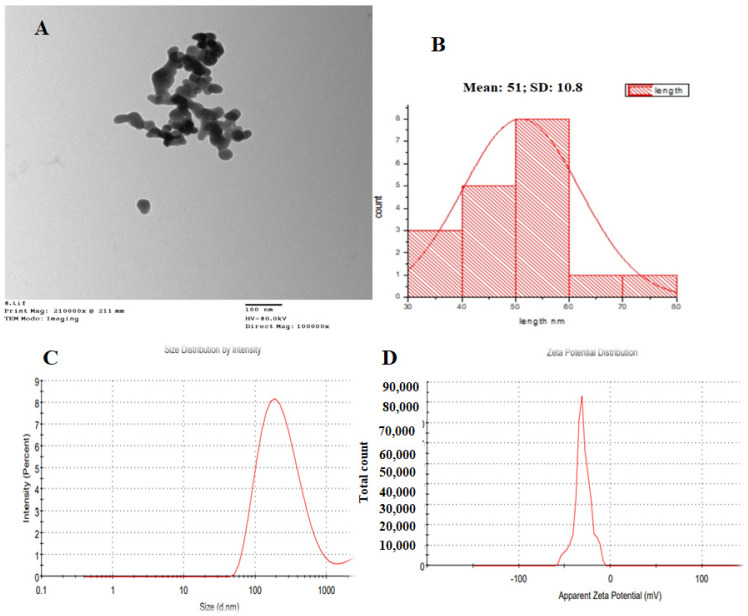
TEM images of SeNPs recorded at (**A**) 100 nm and (**B**) particle distribution, (**C**) Size, and (**D**) Zeta potential pattern of myco-synthesized SeNPs.

The size, form, morphology, and aggregation of SeNPs were investigated using transmission electron microscopy (TEM), which indicated that the particles were equally scattered and had nearly quasi-spherical and spherical shapes with edge lengths ranging from 30 to 78 nm (Figure 3A,B). DLS was utilized to measure the particle size and size distribution of the SeNPs, in addition to their zeta potential. SeNPs had an average hydrodynamic diameter of 213 nm (Figure 3C) and an estimated zeta potential of −30.1 Mv (Figure 3D).

**Figure 4:**
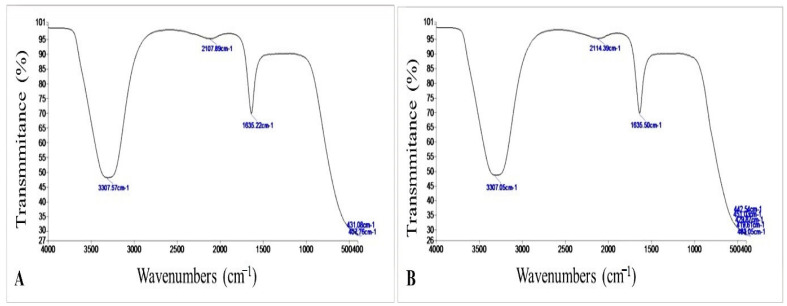

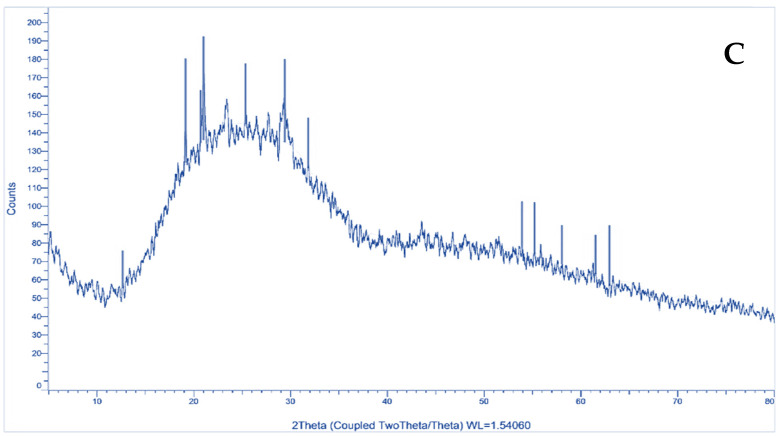
FTIR data of (**A**) *Penicillium chrysogenum* culture filtrate and (**B**) Biosynthesized SeNPs. (**C**) XRD spectrum.

The various functional groups present in the metabolites involved in the myco-synthesis, capping, and stabilization of SeNPs were identified using FTIR studies. The FTIR analysis of *P. chrysogenum* culture filtrate revealed five different peaks at 3307.57, 2107.89, 1635.22, 431.08, and 407.76 cm^−1^ (Figure 4A). In the SeNPs illustration, eight new peaks were added. Figure 4B shows wavenumbers at 3307.05, 2114.39, 1635.50, 451.03, 442.54, 429.87, 419.61, and 403.05 cm^−1^ to indicate the interaction of metabolites with SeNPs. According to the XRD results, myco-synthesized SeNPs appeared less crystalline and more amorphous (Figure 4C).

### 3.2. Antibacterial Activity of SeNPs

#### 3.2.1. Minimum Inhibitory Concentration (MIC)

**Figure 5:**
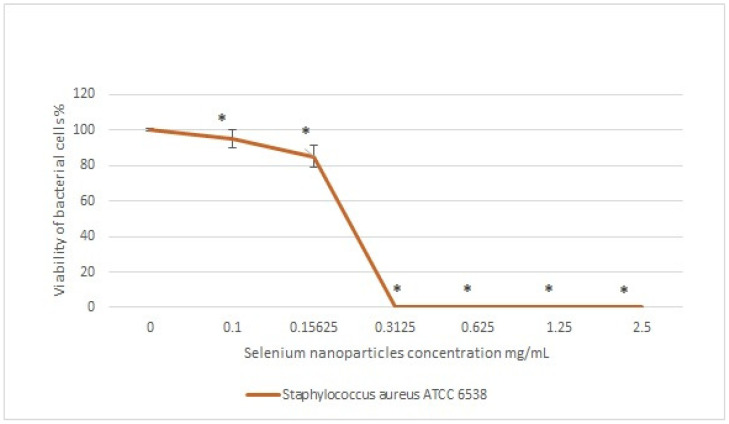
Antibacterial activity and MIC of SeNPs against *S. aureus* ATCC 6538. Data are presented as the mean ± standard error (n = 3). * *p* < 0.05 versus the control group.

Selenium nanoparticles showed antibacterial activity against *Staphylococcus aureus* ATCC 6538, and the activity was clear with increasing SeNP concentration. Furthermore, the percentage of viability of the bacterial strain was zero at a concentration of 0.3125 mg/mL of SeNPs (Figure 5).

#### 3.2.2. Bacterial Morphology Examination by SEM

**Figure 6:**
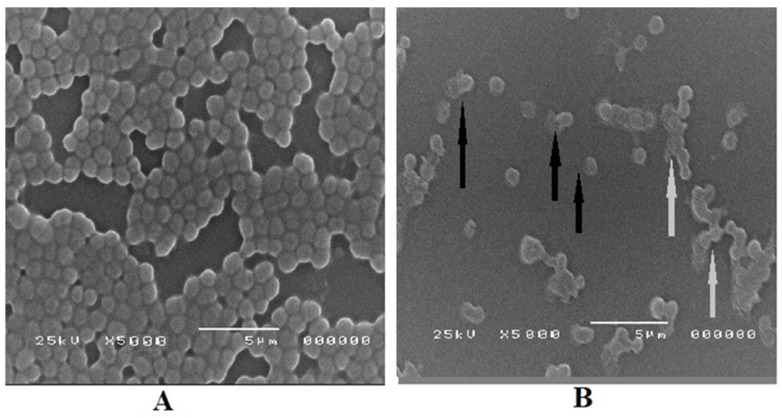
Scanning electron micrograph of *S. aureus* cells morphology. (**A**) Untreated control *S. aureus* cells, while (**B**) *S. aureus* cells treated with SeNPs. Black arrows in B represent internal bacterial cell component leakage resulting from membrane lysis, while grey arrows show irregular and distorted bacterial cells.

The SEM imaging of untreated *S. aureus* bacterial cells showed normal sizes of their structure, regular shapes, and smooth membrane surfaces, as indicated in Figure 6A. In contrast, *S. aureus* cells treated with SeNPs appeared damaged with complete distortion of cells, irregular shapes, partial lysis of membranes, and decreased number of bacterial cells (Figure 6B).

### 3.3. In Vivo Experimental Study

#### 3.3.1. Bacterial Load Determination

**Figure 7:**
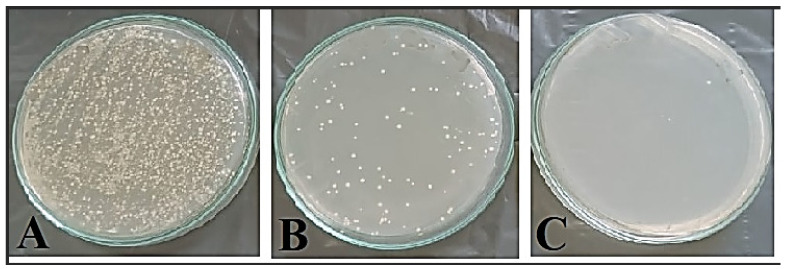
Bacterial load isolated from the prepared wound in different treatments (after six days). (**A**) Not treated (−ve control), (**B**) Treated with gentamicin cream (+ve control), (**C**) Treated with SeNPs.

The bacterial load in the treated tissue with SeNPs achieved the lowest count after six days from the wound treatment compared to the bacterial load in the tissues of positive and negative controls, as shown in Figure 7. The total bacterial count of the wound on zero day was 4 × 10^4^ CFU/mL, but after six days, the total bacterial count was 73 × 10^4^ CFU/mL, 4 × 10^4^ CFU/mL, and 20 CFU/mL in the wounded tissues of negative control, positive control, and treated with SeNPs, respectively.

#### 3.3.2. SeNPs Effects on Wound Diameter

**Figure 8:**
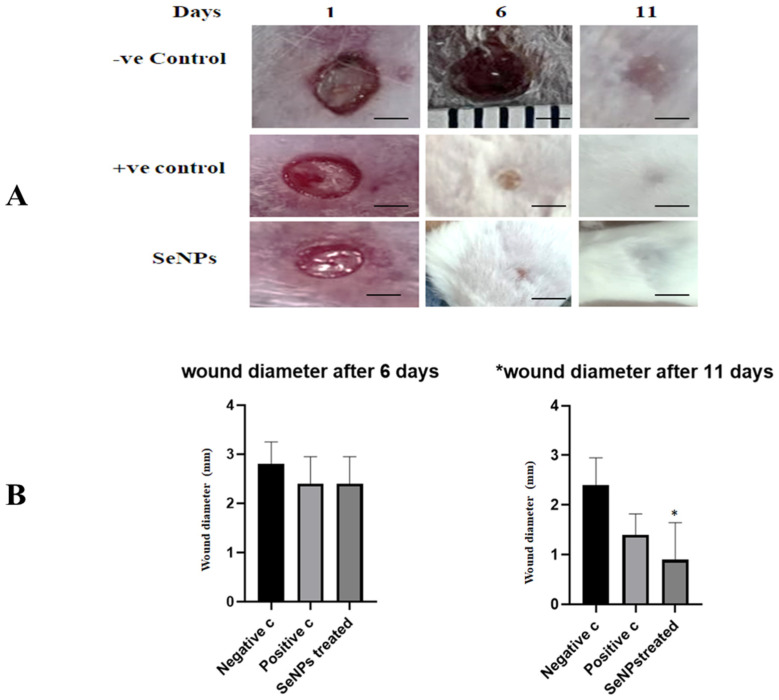
Effect of SeNPs treatment on the closure rate of *S. aureus*-infected wounds. (**A**) Representative photographs of *S. aureus*-infected wounds in −ve control, +ve control, and SeNPs treated groups after six and 11 days of wound injury. (**B**) The wound diameter was measured on days six and 11 of *S. aureus*-infected wounds. Data are shown as mean ± SD (n = 5). Scale bar: 1.6 mm. * is significantly different by one-way ANOVA (*p* < 0.05) compared with negative control.

The treatment with SeNPs could enhance and accelerate the wound healing process, as shown in Figure 8A. There was a significant reduction in the wound diameter of wounded skin treated with SeNPs after 11 days (Figure 8B).

#### 3.3.3. Histological Studies

**Figure 9:**
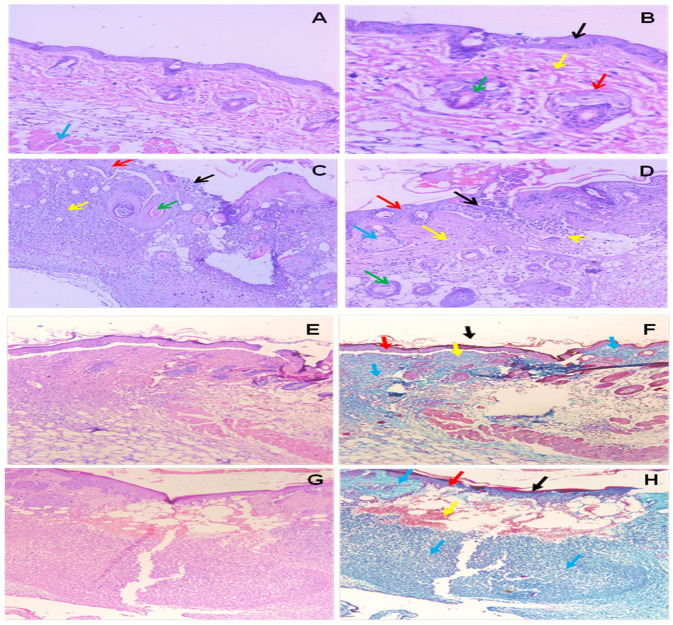
Skin sections from mice; (**A**,**B**) Normal skin with undamaged epidermis (external epithelium formed of 2–3 cell layers) (black arrow), dermis (layer of connective tissue) (yellow arrow), sebaceous glands (red arrow) and hair follicles (green arrow), (piliary canals), muscle layer (blue arrow) (H&E, ×40, ×200). (**C**) A skin section from early +ve mice showed ulcer formation (black arrow), normal skin, intact epidermis (external epithelium formed of 2–3 cell layers) (red arrow), dermis (layer of connective tissue) (yellow arrow), hair follicles (green arrow), (piliary canals), infiltration of epidermis and lymphocytes inflammatory cells (redhead arrows), presence of both sebaceous glands and hair follicles (green arrow) (H&E, ×200). (**D**) A skin section from late +ve control showed ulcer formation (black arrow), normal skin, intact epidermis (external epithelium formed of 2–3 cell layers) (red arrow) and dermis (layer of connective tissue) (yellow arrow), sebaceous glands (blue arrow) and hair follicles (green arrow), (piliary canals), infiltration of the epidermis (black arrows), with a large area with necrosis and granulation tissue (redhead arrow), an absence of both sebaceous glands and hair follicles (yellow head arrow) (H&E, ×200). (**E**,**F**) A skin section from the treated Selenium group showed a healed ulcer (black arrow) with moderate infiltration of the epidermis and dermis by polymorph, neutrophils, and lymphocytes inflammatory cells (blue arrows), absence of both sebaceous glands and hair follicles, intact epidermis (red arrow) and dermis (yellow arrow (H&E, ×100, Masson trichrome ×100). (**G**,**H**) A skin section from the 11-day selenium-treated group showed a healed ulcer (black arrow) with moderately marked infiltration of the epidermis and dermis by polymorph, neutrophils, and lymphocytes inflammatory cells (blue arrows), an absence of both sebaceous glands and hair follicles, intact epidermis (external epithelium formed of 2–3 cell layers) (red arrow) and dermis (layer of connective tissue) (yellow arrow) (H&E, ×100, Masson trichrome ×100).

The current findings demonstrated that a portion of normal mouse skin included a complete epidermis (external epithelium formed of 2–3 cell layers), dermis (layer of connective tissue), sebaceous glands and hair follicles and presence of the biliary canals (Figure 9A,B). The skin of wounded mice (+ve mice) (early stage) demonstrated the development of ulcers accompanied by the infiltration of many polymorphneutrophils and lymphocytes into the epidermis and dermis. Existence of both hair follicles and sebaceous glands (Figure 9C). While at a late stage the ulcer infiltrated the epidermis and dermis by numerous polymorphneutrophils and lymphocytes inflammatory cells, there was a large area with necrosis and granulation tissue with an absence of both sebaceous glands and hair follicles (Figure 9D).

After six days of selenium nanoparticle treatment, the skin section showed a healed ulcer, with moderate infiltration of the epidermis and dermis by polymorph, neutrophils, and lymphocytes inflammatory cells, and an absence of both sebaceous glands and hair follicles (Figure 9E,F). Also, after 11 days of selenium nanoparticles treatment, the skin section showed healed ulcerated with moderately marked infiltration of the epidermis and dermis by polymorph, neutrophils, and lymphocytes inflammatory cells, and an absence of both sebaceous glands and hair follicles (Figure 9G,H).

#### 3.3.4. Proinflammatory Cytokines Detection

**Figure 10:**
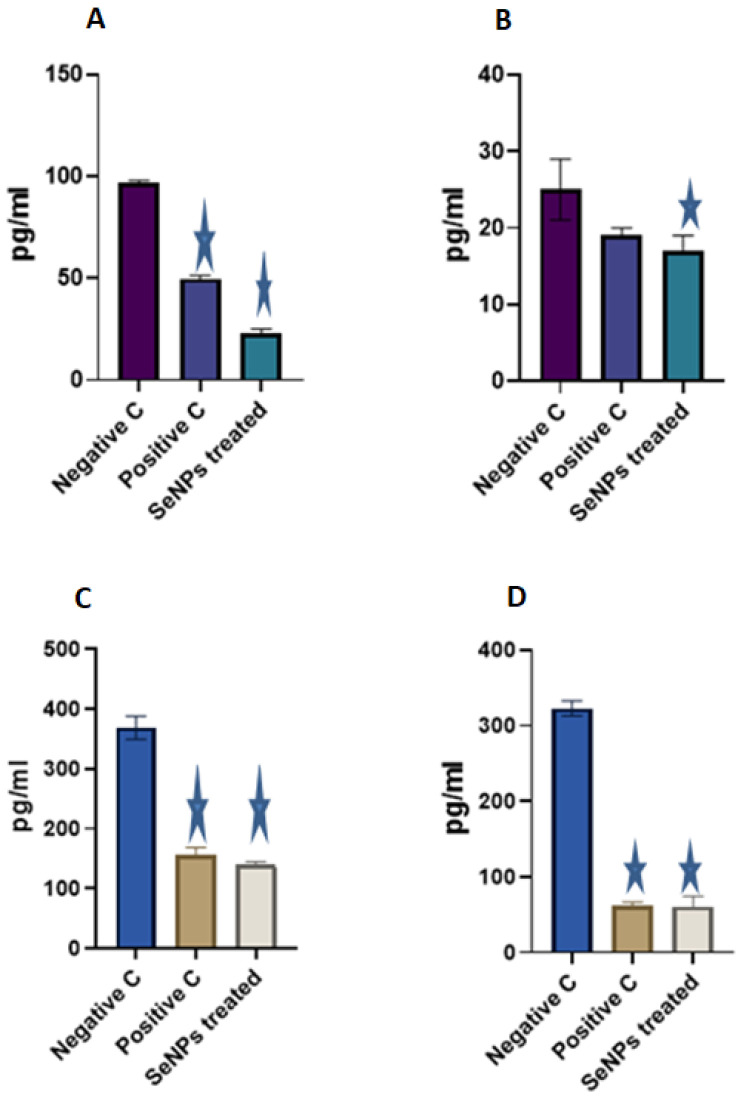
Proinflammatory cytokine levels of mice wounded skin. (**A**,**B**) IL-6 cytokine levels six and 11 days post wound. (**C**,**D**) TNF-α cytokine level six and 11 days post wound. Data are provided as means ± standard deviations and compared using one-way ANOVA (n = three mice per group per experiment). 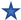 is significantly diferent by one-way ANOVA (*p* < 0.05) compared with negative control.

SeNPs showed a promising effect on the proinflammatory cytokines of mice’s wound skin. ELISA readings showed a significant reduction in IL-6 after treatment with SeNPs for six days (23 pg/mL) and 11 days (17 pg/mL) days and TNF-α (134 pg/mL and 61 pg/mL) for six and 11 days of treatment, respectively, as compared to the negative controls (Figure 10).

### 3.4. Docking Study

**Figure 11:**
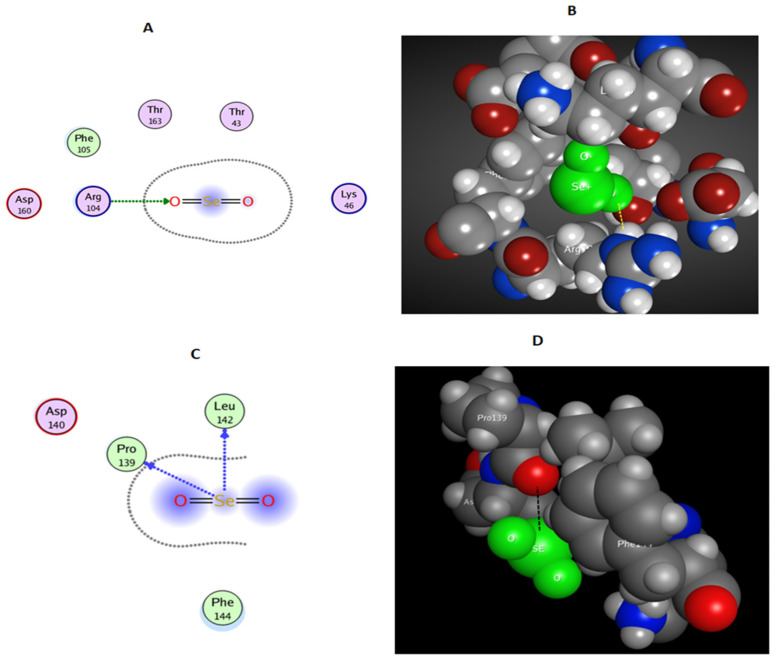
A docked 2D and 3D interaction map for the SeNPs with the proinflammatory cytokines IL-6 (**A**,**B**) and TNF-α (**C**,**D**).

The effects of selenium nanoparticles on the inflammatory cytokines IL-6 and TNF-α were studied (SeNPs). Docking experiments showed that selenium nanoparticles were very effective at competing with the binding sites of IL-6 (Figure 11A,B) and TNF-α (Figure 11C,D) receptors. The interaction-free energy was employed to investigate the influence of the ligand on both cytokines. Selenium nanoparticles were able to dock with these cytokines through their H-interaction scores (−2.2 Kcal/mol) for IL-6 and (−1.7 and −0.4 Kcal/mol) for TNF-α (Table 1).

## 4. Discussion

Staphylococcal skin infections are a prevalent problem in both chronic wounds and surgical sites, and they significantly impede the healing process [33]. *Staphylococcus aureus* is a major public health concern due to rising antibiotic resistance [34], and methicillin-resistant *S. aureus* (MRSA) is a therapeutic obstacle due to its significant involvement in delaying wound healing [35]. Selenium nanoparticles have been shown to have antibacterial and wound therapeutic properties [36]. In this investigation, the green synthesis of SeNPs was employed by *P. chrysogenum* culture supernatant metabolites. *P. chrysogenum* may create a variety of extracellular biomolecules, such as carbohydrates, enzymes, and proteins, that function as biological catalysts in the reduction and stabilization of nanoparticles [21,37]. After incubation, synthesized SeNPs were removed from the culture medium and purified before being characterized using a variety of procedures. *P. chrysogenum*-mediated SeNPs displayed UV-maximum absorption peaks at 520 nm in the UV spectrum. This peak might be due to the influence of the SeNPs surface plasmon resonance [21,22]. It was stated that the absorbance measurement for SeNPs produced by some endophytic fungi was obtained at 265 [22]. It has also been observed that SeNPs have absorption spectra at 300 nm and 540 nm [38,39]. The diameters estimated by DLS (213 nm) and TEM (30 to 78 nm) varied because DLS determines the hydrodynamic size, whereas TEM checks the solid center [19]. According to Vahidi et al. [40], the negative charge of particles reflects the electrostatic stability of the synthesized nanoparticles. The NPs’ stabilizing negative charges are due to the fungus protein that coats their surface [41]. The large difference in size observed between DLS and TEM measurements could be attributed to the SeNPs aggregation. Nanoparticles, when synthesized, exhibit a high reactivity due to the presence of a significant number of dangling bonds and defects on their surfaces [42]. Small grain sizes result in high surface energy, and mechanisms to lower surface energy through NP assembly might initially lead [42]. In our investigation, the thermodynamic stabilization of these nanomaterials appeared to be caused by the organic molecules surrounding the biogenic SeNPs. Any modifications made to these molecules during the washing and drying processes could affect the electrosteric barrier and increase the energy and reactivity of the nanoparticles, which would then lead to their aggregate formation and fix their thermodynamic instability [43]. A zeta potential larger than +30 mV or a value lower than +30 mV suggests the colloidal nanoparticle solution electrostatic stability [44]. According to the XRD data, the myco-synthesized SeNPs appeared amorphous. This amorphous form of selenium nanoparticles corresponds to prior findings using *Pseudomonas stutzeri* [45], lycopene [46], and *Withania somnifera* [47]. The data obtained in the present study demonstrated that the ultraviolet-visible spectrum of SeNPs exhibited a peak absorbance at a wavelength of 520 nm. Furthermore, the structural examination of the SeNPs synthesized through *P. chrysogenum* metabolites indicated that they had a spherical shape, with sizes ranging from 30 to 80 nm and an estimated zeta potential of −30.1 Mv. Additionally, these nanoparticles were shown to possess an amorphous structure. Further study was required for more and deeper characterization.

The interaction of *P. chrysogenum* extracellular compounds and SeNPs was discovered via FTIR investigation. The signal band at 3307 cm^−1^ represents N-H, C-H, and O-H stretching vibrations, indicating the presence of primary amine [48], alkyne, and alcohol, respectively [49]. This highlights the significance of N-H-containing proteins in the production of SeNPs. The presence of alkyne is shown by a band at 2114 cm^−1^. Furthermore, the signal at 1635.50 cm^−1^ was linked to peptide bonding and polysaccharide ring constituents such as N-H, C=N, C=O, and C=C [50].

In the current study, in vitro and in vivo studies were carried out to determine the antibacterial and wound healing features of the myco-synthesized SeNPs against *Staphylococcus aureus* ATCC 6538. The generated SeNPs clearly inhibited *S. aureus* at a MIC value of 0.3125 mg/mL, as shown in vitro. Additionally, the antibacterial activity was confirmed in the in vivo results on SeNP-treated wounds by the reduction of the total bacterial load comparing wounds with positive and negative controls. Salem [19] reported that the MIC values for SeNPs against *Escherichia coli*, *Staphylococcus aureus*, *Aspergillus fumigatus*, and *Aspergillus niger* were 62.5, 125, 250, and 500 µg/mL, respectively. Also, Vahdati and Moghadam [51] determined that 82 µg/mL of SeNPs was sufficient to suppress the growth of *S. aureus*. Eleraky et al. [52] reported that the antimicrobial activity of nanoparticles could be caused in several different ways, such as the release of reactive oxygen species (ROS), inhibition of protein and DNA synthesis, activation of metabolic genes, disruption of cell walls, and membrane permeability. Moreover, the antimicrobial action in metal-based nanoparticles is commonly associated with the formation of reactive oxygen species (ROS) (hydroxyl radicals, superoxide anions, and hydrogen peroxide). In addition to causing damage to the bacterial cell membrane, reactive oxygen species can impede DNA replication and amino acid synthesis [53]. SEM data showed that cells treated with SeNPs appeared deformed, with abnormal shapes and lysed membranes, and the total number of cells decreased. Further study was required to confirm the penetration or aggregation of SeNPs in the bacterial cell, and the inhibitory effect and morphological changes on *S. aureus* cells were observed after the overnight incubation of the bacterial cells with the nanoparticles alone in the same conditions as the control. These results were explained by Wang et al. [54], who demonstrated that NPs are more effective against Gram-positive bacteria than Gram-negative bacteria because the cell wall of Gram-negative bacteria is made up of lipopolysaccharides (LPS), lipoproteins, and phospholipids, which form a penetration barrier that only enables the entry of macromolecules. In contrast, the cell wall of Gram-positive bacteria has a thick layer of peptidoglycan, teichoic acid, and numerous pores that let foreign molecules in, causing damage to the cell membrane and cell death. Additionally, Gram-positive bacteria have a higher surface negative charge than Gram-negative bacteria, which can attract nanoparticles (NPs). Selenium has the potential to bind to the surface of cells, interfering with their permeability and their ability to breathe. SeNPs can likely do more than only interact with membranes on the outside of bacteria; they can also get inside and kill them [55]. It is important to assess the histological status of the wound healing process for postoperative patient management [56,57]. Van De Vyveret et al. [30] stated that histopathological analysis of the wound autopsies could provide significant insight into healing dynamics. The examination should encompass the fundamental parts of healing, and the degree of change noticed should be scored as a semi-quantitative score, namely, low, medium, or high grade [57]. The present results showed that the skin sections from the SeNPs treated group, either from six or 11 days, exhibited a healed ulcer with mild infiltration of the epidermis and dermis by polymorph, neutrophils, and lymphocytes, the lack of sebaceous glands and hair follicles, and an otherwise intact epidermis and dermis. These results were in good accordance with Santos et al. [58], who reported that hydroalcoholic extract of *Vitis labrusca* (HEVL) oral administration stimulated the formation of interstitial collagens, fibronectin, and elastin in response to growth factors generated by macrophages, which sped up the healing of injured tissue, especially after 14 days [59]. Also, Khalaf et al. [29] indicated that early polymorph infiltration was identified at 4–7 h following skin wound tissue section histological investigation, with peak levels at 24-h, the granulation tissue formation is seen by five to eight days, and the collagen formation begins at three to six days and increases in density after 14 days. More investigation was required to validate the penetration of nanoparticles into epithelial cells.

The most important step of the healing process for wounds is the inflammatory phase. Prolonged inflammation, on the other hand, causes increased cytokine production, such as IL-1, IL-6, and TNF-, severe healing disturbances, and increasing fibrosis and scarring [60]. TNF-α is a proinflammatory cytokine expressed in higher levels during the inflammatory stages than at other times [61]. Additionally, IL-6 functions as a multifunctional cytokine with pleiotropic effects on hematopoiesis, immunological responses, and inflammation [62]. The present study revealed that SeNPs could decrease both cytokines, IL-6, and TNF-α levels at six and 11 days after wound exposure compared with the control group. Similar data were observed by Nguyen et al. [63], who found that calophyllolide compound extracted from *Calophyllum inophyllum* Linn. might be effectively applied for wound healing in a murine mouse model, as it could regulate the inflammatory cytokines response through reduction of some proinflammatory cytokines such as IL-1β, IL-6, and TNF-α. Moreover, Pereira et al. [64] concluded that the natural triterpene lupeol, which is a bioactive found in various edible plants, might be used as a strong wound-healing agent as they detected a reduction in the levels of the proinflammatory cytokines (TNF-a, IL-1, and IL-6) after 7 and 14 days. In several pathologic processes involving inflammation and tissue deterioration/remodeling, elastase and melloprteinase (MMP9) activity have been identified as crucial factors. Patients suffering from acute wounds have higher levels of inflammatory cell-derived MMPs in their tissues, higher levels of MMP9 in their wound fluid, and lower levels of protease inhibitors. TNF-α induces expression of MMP9 in various cell types, so blocking the TNFα pathway would benefit the delayed healing model. After TNF activates TNFR1p55/TNFR2p75, adaptor proteins are recruited to start signaling pathways that lead to the transcription of inflammatory genes. TNF-α promotes NFκB, which in turn triggers gene production of numerous proinflammatory cytokines, including TNF and proteases like MMP, to release soluble TNF, amplifying the effects of this inflammatory cytokine. The key mechanism to reduce TNF-α is the blockage of NFκB activity [65]. The delayed healing model suggests that inducible nitric acid synthase, MMP9, and TNF are abundantly expressed by macrophages and are probably due to NFκB activation, which worsens the inflammatory response to tissue injury [66].

Moreover, wound healing may be impacted by modifying the IL-6 signaling pathway. Activating the Angiotensin 2 receptor blocked the expression of IL-6, TNF-, and TGF-β, which showed antiinflammatory effects [67]. The down-regulated IL-6 expression can be achieved by inhibiting NF-κB and the STAT3 and smad3 signal transduction pathways [68].

Understanding and predicting the activity of the ligand complex SeNPs against some proteins through receptor-ligand interactions is possible through molecular docking. The production of cytokines is a crucial stage in macrophages’ reaction to inflammatory stimuli [69]. Macrophages are an important source of several cytokines and growth factors activated by foreign particles. Interestingly, uncontrolled inflammatory response can result in severe chronic inflammation [70]. Macrophages release several inflammatory molecules, including tumor necrosis factor-α (TNF-α), interleukin-6 (IL-6), and IL-1β, through which the inflammatory process can be regulated [71]. The present investigation showed an interaction between the ligand, SeNPs, and IL-6 proteins that may result in the reduction of levels of this cytokine. Although TNF-α is a crucial component regulating wound healing, chronic TNF overexpression impairs skin regeneration, and patients treated with TNF inhibitors consistently exhibit delayed skin regeneration [72]. Additionally, the molecular docking demonstrated a possible interaction between SeNPs and TNF-peptide, which could be a good sign for lowering TNF-α levels.

## 5. Conclusions

This study showed that the green fabrication of selenium nanoparticles by *Penicillium chrysogenum* MZ945518 was antibacterial against *S. aureus* ATCC 6538 and helped heal wounds. In vitro results showed antibacterial properties with increasing SeNPs concentration, and the MIC value recorded was 0.3125 mg/mL. In addition, the inhibition effect was investigated by determining the bacterial load of the wounded tissues through the in vivo experiment, and the results showed a clear reduction of the bacterial count from 73 × 10^4^ CFU/mL in the negative control to 20 CFU/mL in the treated tissues with selenium nanoparticles. SEM images of *S. aureus* cells under nanoparticle treatment showed complete distortion of cells, irregular shapes, partial lysis of membranes, and a decreased number of bacterial cells. Furthermore, an in vivo evaluation of wound healing capacity revealed that SeNPs showed potential wound healing effects in mice by speeding up the wound closure rate and decreasing the levels of cytokines IL-6 and TNF-α, in addition to the histopathological investigation that revealed healing of wounded tissue treated with SeNPs and the formation of collagen. As a result, this study demonstrates that myco-synthesized SeNPs might be an efficient candidate for treating *S. aureus* wound infections and accelerating wound healing.

## Figures and Tables

**Table 1:** *In-silico* docking study of the ligand SeNPs with the proinflammatory cytokines IL-6 and TNF-α.

PDB ID	Docking Score (Kcal/mol)	Interaction Type	Amino Acid Residue
IL-6 (1alu)	−2.2	H-acceptor	ARG 104
TNF-α (2az5)	−1.7 −0.4	H-donor H-donor	PRO 139 LEU 142

Abbreviations ARG: Arginin amino acid; LEU: Leucine amino acid; PRO: Proline amino acid.

## Data Availability

All data generated or analyzed during this study are included in this article.
